# Traction‐Induced Avulsion of an Iliocaval Venous Branch During Multilevel OLIF for Degenerative Scoliosis: Case Report

**DOI:** 10.1002/ccr3.73011

**Published:** 2026-09-29

**Authors:** Tetsuro Ohba, Nobuki Tanaka, Kotaro Oda, Hayato Takei, Kai Mizukami, Go Goto, Hirotaka Haro

**Affiliations:** ^1^ Department of Orthopaedic Surgery University of Yamanashi Yamanashi Japan

**Keywords:** adult degenerative scoliosis, case report, inferior vena cava, lateral lumbar interbody fusion, vascular injury, venous avulsion

## Abstract

Adult degenerative scoliosis can be effectively treated with lateral or oblique lumbar interbody fusion (LLIF/OLIF), but vascular injury remains a rare and potentially catastrophic complication. We report the case of a 78‐year‐old woman with long‐standing degenerative scoliosis who underwent multilevel OLIF from L2 to L5. During trial cage placement at L2–3, massive venous hemorrhage occurred, with an estimated blood loss of approximately 3750 mL. Intraoperative angiography showed no arterial extravasation, suggesting a venous source. The wound was packed, and the patient was stabilized with aggressive resuscitation. Reexploration 3 days later, performed with a vascular surgeon, identified an avulsed iliocaval venous branch at the anterior aspect of the L2 vertebral body. Hemostasis was achieved using a fibrin sealant patch and sustained compression. After recovery, staged posterior fixation was completed without further complication. The patient recovered without neurologic deficit and maintained satisfactory alignment at follow‐up. This case highlights a rare indirect vascular injury mechanism during deformity correction, likely caused by traction on a stiff iliocaval venous branch during multilevel distraction. Careful preoperative vascular assessment, gradual disc‐space distraction, readiness for staged management, and early vascular surgery involvement are essential to improve safety in complex OLIF procedures.

## Key Clinical Message

Multilevel OLIF for adult degenerative scoliosis can cause catastrophic traction‐induced avulsion of an iliocaval venous branch, even without direct instrument injury. Careful preoperative vascular assessment, gradual disc‐space distraction, prompt packing, and early vascular surgeon involvement are essential for safe management. This staged, multidisciplinary strategy can preserve correction and patient safety.

## 1 Introduction

Adult spinal deformity surgery can greatly improve pain and function but entails nontrivial perioperative risks [1]. Lateral and oblique lumbar interbody fusion (LLIF/OLIF) provides minimally invasive anterior access for disc‐height restoration and segmental realignment, yet major vessels lie in close proximity to the operative field [2, 3, 4]. Although vascular complications are infrequent, they remain potentially devastating, with reported injuries ranging from segmental vessels to the left common iliac vein [5, 6, 7]. Preoperative MRI‐ or CT‐based risk stratification—particularly at L4–L5—helps estimate vessel proximity and corridor width and can guide side selection [4]. Beyond direct laceration by instruments or retractors, aggressive deformity correction itself may impose tensile loads on the vasculature; indeed, abdominal aortic lengthening has been observed following multilevel realignment, implying traction stress [8]. To our knowledge, traction‐induced avulsion of an iliocaval venous branch during multilevel OLIF has rarely, if ever, been documented, making this event an exceptionally uncommon complication. Here, we describe a suspected traction‐induced avulsion of an iliocaval venous branch during multilevel OLIF and outline preventive strategies and management principles.

## 2 Case History and Examination

A 78‐year‐old woman with long‐standing degenerative lumbar scoliosis (Cobb angle ~50°) presented with disabling back pain and neurogenic claudication refractory to conservative measures. Comorbidities included hypertension; there was no history of coagulopathy, collagen disorders, or prior abdominal surgery. Standing full‐length radiographs showed coronal malalignment and fixed thoracolumbar kyphosis. Contrast‐enhanced planning CT in lateral decubitus demonstrated the left common iliac artery immediately ventral to L5–S1, prompting us to avoid an L5–S1 OLIF approach and plan OLIF at L2–L5 followed by staged posterior fixation (Figure 1A–C).

**FIGURE 1:**
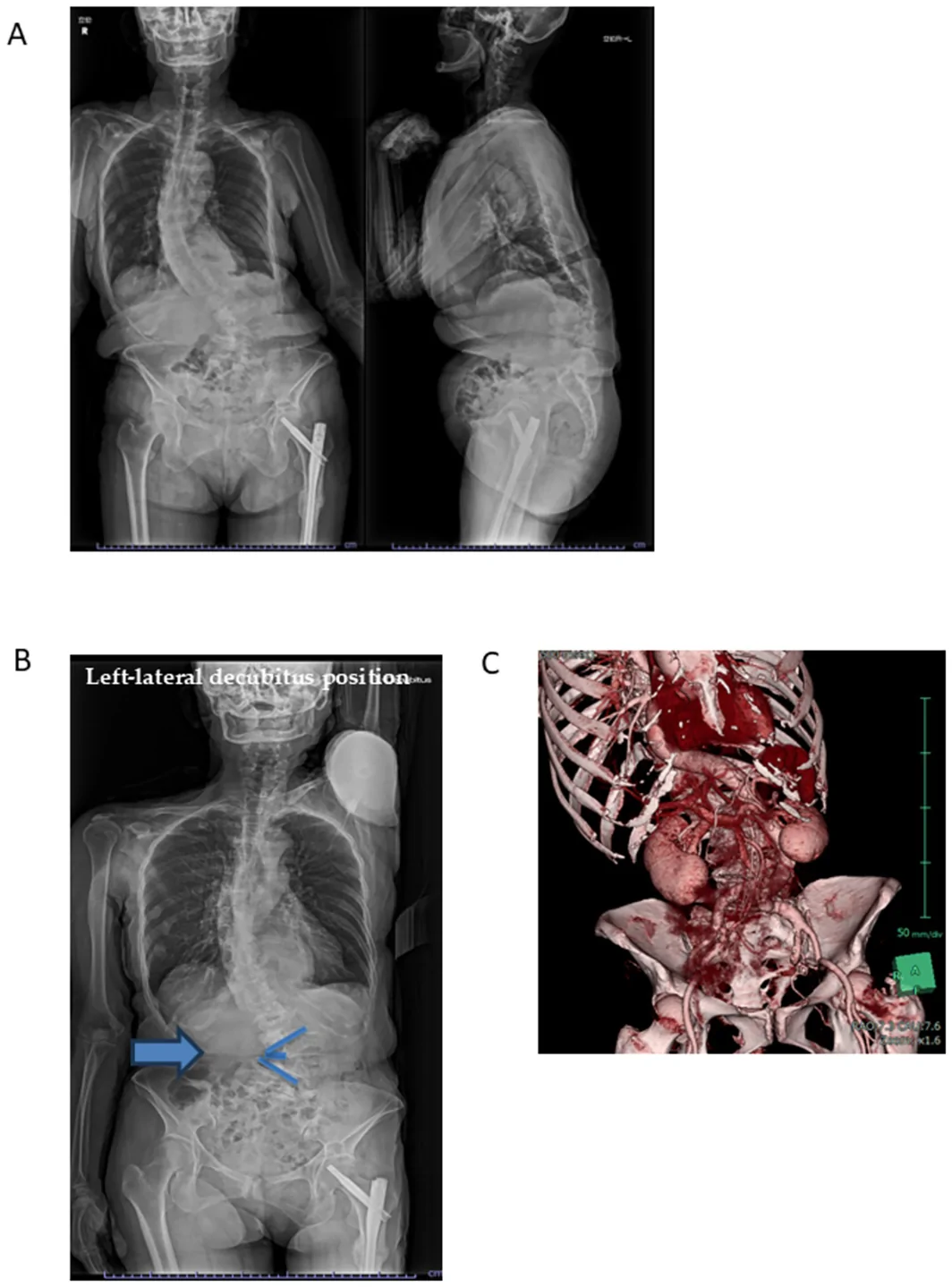
Preoperative imaging. (A) Standing whole‐spine radiograph showing degenerative lumbar scoliosis with coronal malalignment and fixed thoracolumbar kyphosis. (B) Contrast‐enhanced planning CT in the lateral decubitus position demonstrating the left common iliac artery immediately ventral to L5–S1, leading to avoidance of the L5–S1 OLIF approach. (C) Three‐dimensional reconstruction of contrast‐enhanced CT showing the vascular anatomy and its relationship to the planned OLIF corridor.

Intraoperatively, the patient was positioned in left lateral decubitus. An O‐arm navigation frame was secured to the L3 spinous process. The retroperitoneal corridor was developed through sequential dilation; navigation confirmed mid‐disc access. Discectomy and lateral osteophyte release were performed at L4–5, L3–4, then L2–3. Low‐profile cages were selected to avoid over‐distraction; blood loss was minimal at the first two levels.

Upon trial insertion at L2–3, we observed slow oozing from the anterior disc space, followed by a sudden gush of dark venous blood upon removing the trial. Estimated blood loss rapidly exceeded 3.5 L. The final estimated blood loss for the anterior stage was approximately 3750 mL, estimated from suction canister volume after subtracting irrigation fluid, together with blood‐soaked gauze and temporary packing. Hemostatic patties were applied immediately and held with firm pressure. There were no malpositioned instruments on fluoroscopy. On‐table angiography via femoral access showed no arterial extravasation; empiric embolization of the L3 lumbar artery did not reduce bleeding, suggesting a venous source (Figure 2A). The wound was tightly packed with gauze and temporarily closed. Aggressive resuscitation with crystalloids and blood products including packed red cells restored hemodynamic stability (Figure 2B,C). Perioperative resuscitation details were as follows: packed red blood cells 14 units, fresh frozen plasma 10 units. Hemoglobin was 13.5 g/dL preoperatively, 8.4 g/dL immediately after the anterior stage, and 11.4 g/dL before reexploration. Coagulation studies showed PT‐INR 1.2, APTT 31 s, fibrinogen 340 mg/dL, and platelet count 280 × 10^9^/L.

**FIGURE 2:**
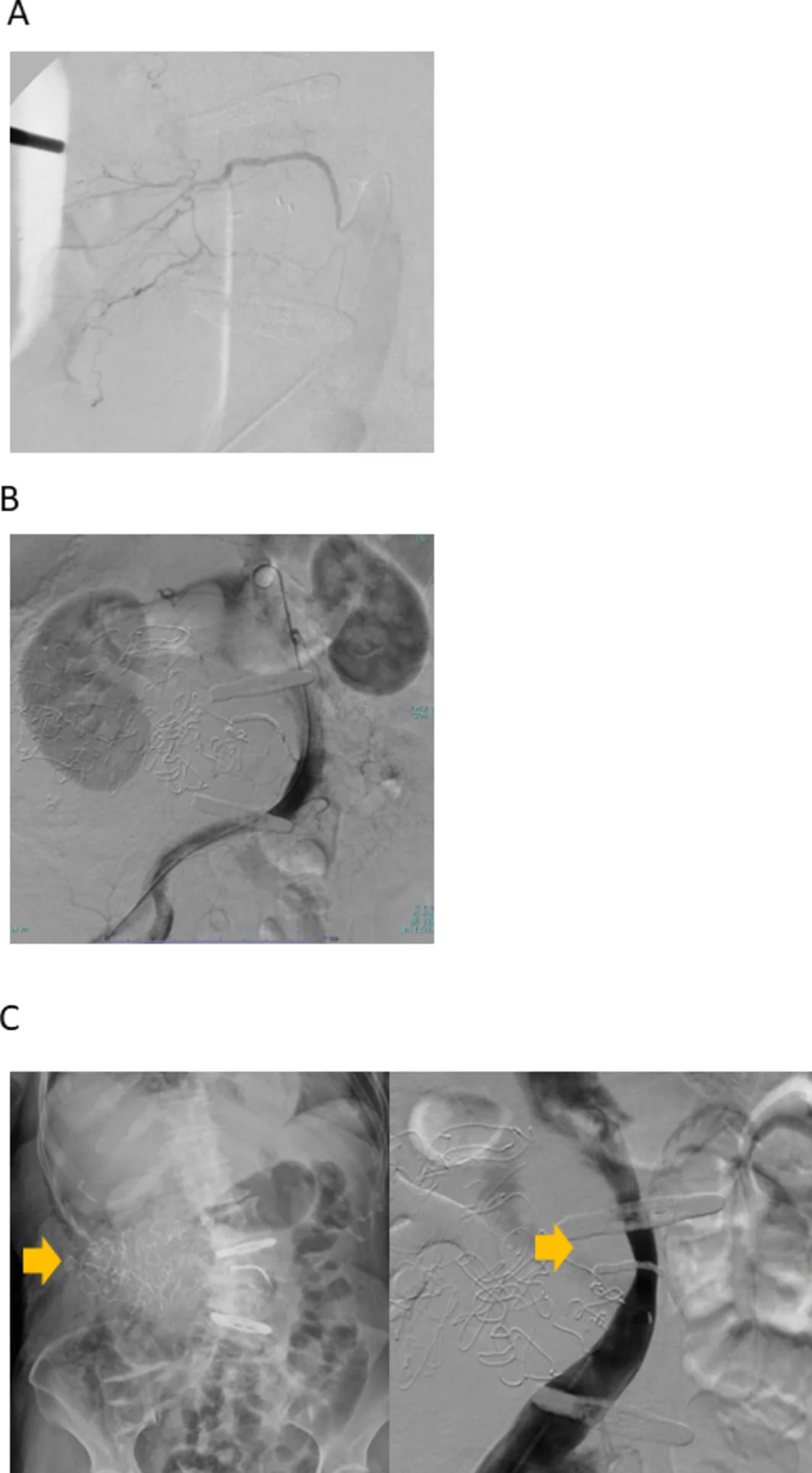
Intraoperative hemorrhage during OLIF at L2–3 and initial management. (A) Intraoperative angiography showing no arterial extravasation or arterial blush. (B) Intraoperative view showing sudden dark venous bleeding from the anterior disc space after removal of the trial cage. (C) Temporary hemostasis was achieved by firm compression with gauze packing. The yellow arrow indicates the gauze used for direct compression of the bleeding site.

On postoperative Day 3, with a vascular surgeon present, we reopened the retroperitoneal approach. Removing the packs revealed a persistent oozing point at the anterior L2 body. Closer inspection identified an avulsed venous tributary from the iliocaval plexus, likely a perivertebral vein torn off the vena cava. Definitive hemostasis was achieved with a fibrin sealant patch applied directly over the lesion plus sustained compression; no ligation was required. After 72 h of surveillance, hemoglobin stabilized without further bleeding or thrombotic events (Figure 3A,B).

**FIGURE 3:**
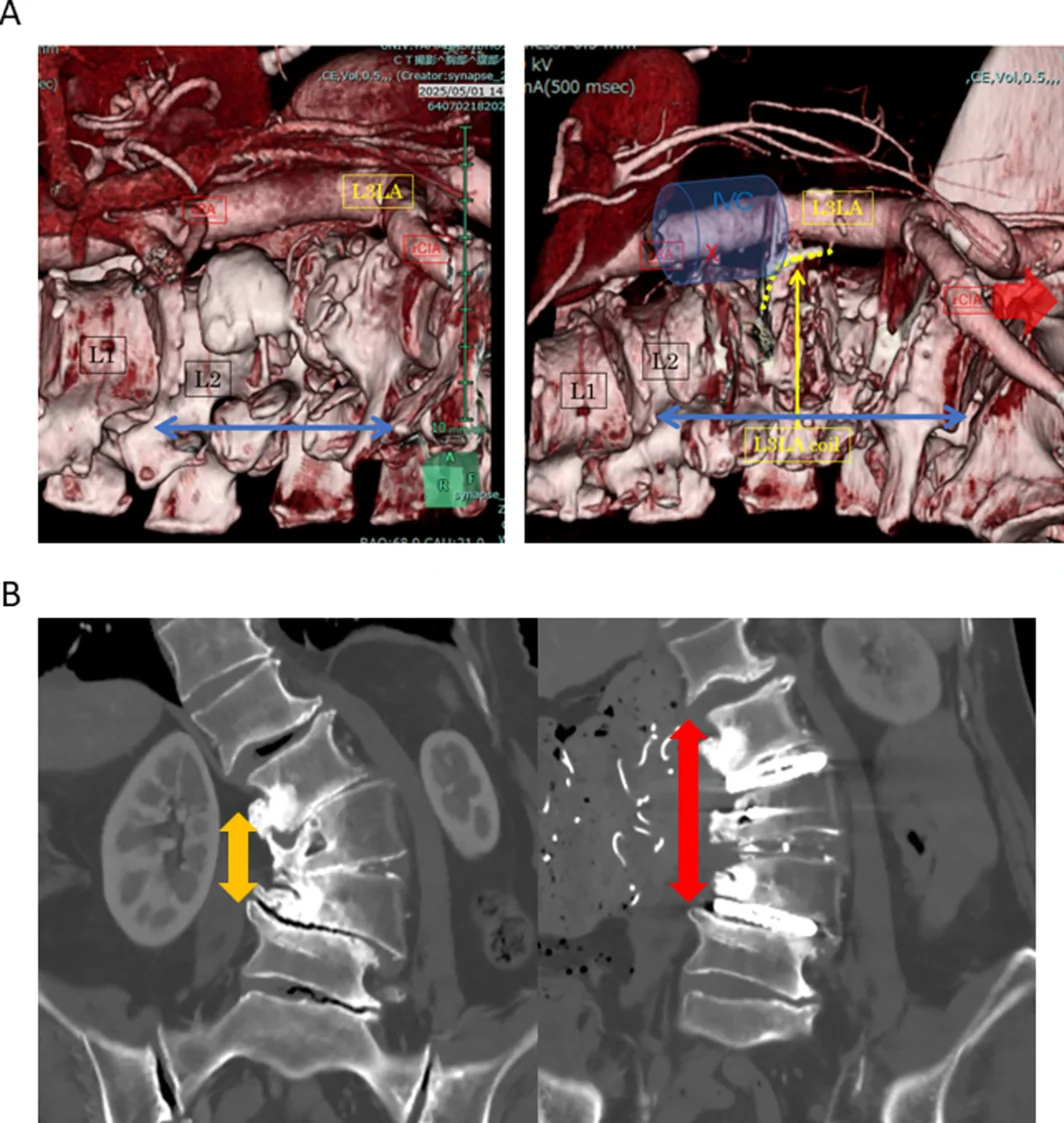
Reexploration and definitive hemostasis. (A) Reexploration on postoperative day 3 showing an avulsed perivertebral venous branch at the anterior aspect of the L2 vertebral body. (B) Comparison of preoperative and postoperative CT images showing elongation of the concave side after scoliosis correction. The arrows indicate the concave‐side elongation, suggesting traction stress on the adjacent vascular structures.

## 3 Outcome and Follow‐Up

Three weeks later, once the patient had recovered from the anterior‐stage insult, she underwent staged posterior instrumentation and fusion from T10 to the pelvis with neuromonitoring. Sagittal realignment was restored with careful rod contouring; estimated blood loss was 800 mL. The postoperative course was uneventful. At 3 months, the patient ambulated independently, reported resolution of radicular pain, and maintained satisfactory alignment on radiographs (Figure 4). The available follow‐up duration was 3 months after posterior fixation. During this period, no recurrent retroperitoneal hemorrhage, thrombotic event, wound complication, or neurological deterioration was observed.

**FIGURE 4:**
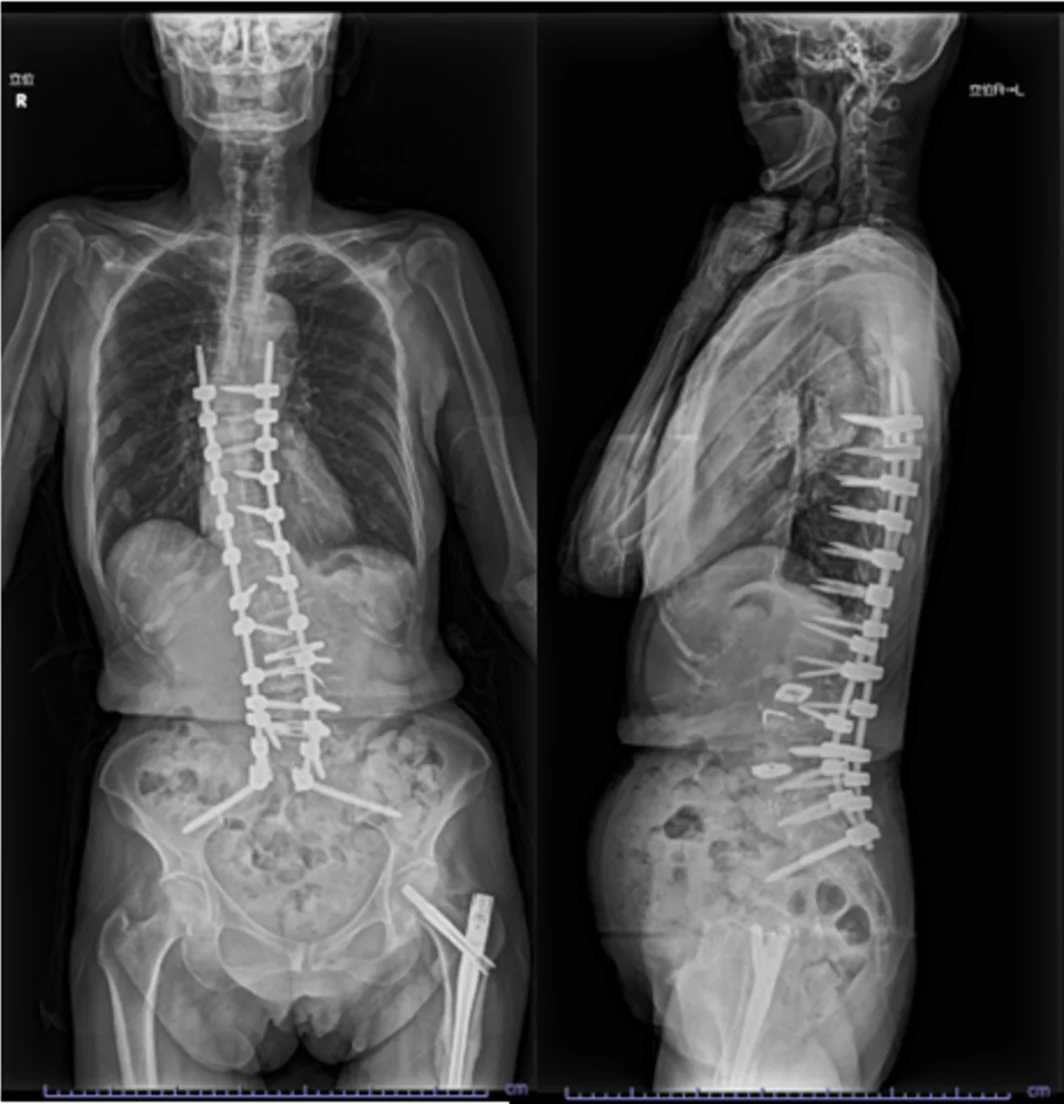
Postoperative radiographs. Standing radiographs obtained 3 months after staged posterior fusion showed satisfactory coronal and sagittal alignment.

## 4 Differential Diagnosis

Initial intraoperative differential diagnoses for sudden hemorrhage included:

- Arterial injury (e.g., lumbar segmental artery, common/iliac arteries).
- Venous injury (e.g., perivertebral/iliocaval tributary avulsion or laceration).
- Coagulopathy‐related diffuse bleeding.

Absence of an arterial blush on angiography supported a venous source, later confirmed as traction‐induced avulsion of an iliocaval branch during multilevel OLIF.

## 5 Discussion

LLIF/OLIF allows substantial coronal and sagittal correction with limited muscular disruption; however, the vasculature—particularly the inferior vena cava and iliac veins—courses immediately anterior to the L2–L5 vertebral bodies. Published series report low overall vascular injury rates, but major events, when they occur, disproportionately involve the left common iliac vein [6, 9, 10, 11]. Venous injuries can produce brisk hemorrhage yet be angiographically occult, leading to diagnostic uncertainty and delayed targeted hemostasis.

Our case illustrates an indirect (traction‐type) pathomechanism. Progressive multilevel distraction—especially when L2–3 is addressed after L3–4 and L4–5—may cumulatively stretch ventral perivertebral veins. In elderly patients, vessel stiffening and preexisting tortuosity likely lower the failure threshold of venous branches. The observation that the abdominal aorta lengthens measurably after deformity correction supports the concept that the great vessels experience significant tensile loads during spinal realignment [8]. Alternative explanations include an occult direct venous wall injury during discectomy or trial insertion, retractor‐related compression or shear, tearing of a small perivertebral vein during anterior osteophyte release, or coagulopathy‐related diffuse bleeding. These possibilities were considered; however, the absence of malpositioned instruments, negative arterial angiography, and the focal avulsed tributary identified at reexploration favored traction‐induced avulsion.

Risk mitigation begins preoperatively [12, 13]. Contrast‐enhanced MR/CT should be scrutinized for corridor width, vessel‐to‐disc distance, and variants (e.g., anomalous iliac veins) at each target level [12, 14, 15]. Side selection should reflect the safest corridor [13]. Intraoperatively, surgeons should employ stepwise releases, select modest cage heights, and avoid sudden distraction [13]. When bleeding occurs without an arterial blush on angiography, early consideration of venous avulsion is warranted. Packing and staged return with a vascular surgeon, as in this case, can be lifesaving.

Team‐based perioperative management is equally important. Massive blood loss mandates rapid activation of transfusion protocols, vigilant hemodynamic monitoring, and postoperative surveillance for coagulopathy or thrombosis. Staged posterior correction after recovery minimizes physiologic stress and allows safer completion of deformity surgery. Ultimately, anticipating traction‐related vascular complications may reduce morbidity without compromising radiographic correction.

## 6 Conclusion

Traction‐induced avulsion of an iliocaval vein branch during multilevel OLIF is rare but catastrophic [16]. Detailed preoperative vascular mapping, incremental distraction, readiness to abort and pack the anterior stage, and early involvement of vascular surgery are key safeguards [13, 16]. With these measures, complex deformity corrections can proceed with improved safety.

## Author Contributions

**Tetsuro Ohba:** conceptualization, data curation, formal analysis, investigation, methodology, project administration, supervision, validation, writing – original draft, writing – review and editing. **Nobuki Tanaka:** methodology, supervision, validation. **Kotaro Oda:** data curation, investigation, methodology, software, validation, visualization. **Hayato Takei:** data curation, investigation, validation. **Kai Mizukami:** data curation, formal analysis. **Go Goto:** data curation, formal analysis, methodology. **Hirotaka Haro:** resources, supervision, validation.

## Funding

The authors have nothing to report.

## Ethics Statement

The Institutional Review Board of the University of Yamanashi waived approval for this single‐patient report.

## Consent

Written informed consent for publication was obtained from the patient.

## Conflicts of Interest

The authors declare no conflicts of interest.

## Data Availability

The data that support the findings of this study are available on request from the corresponding author. The data are not publicly available due to privacy or ethical restrictions.
